# History of Dentistry

**Published:** 1887-04

**Authors:** 


HISTORY OF DENTISTRY.—Dr. E. P. Beadles in answer to a question pertaining to the early history of dentistry replied as follows:

Herodotus, 500 B. C., speaks of physicians for the teeth. The Egyptians were somewhat advanced in the science, as many filled teeth are found in the mouths of mummies. Celsus, who lived 100 B. C., recommended certain dental operations. So remote is the origin of dental surgery, and imperfect the history of ancient medicine, that we cannot trace it with any degree of certainty.

It was not until 1728 that a systematic treatment on Dental Surgery was published; this was a book of some nine hundred pages, written by Pierre Fanchard. Then followed works by Burou, Lecluse, Jourdain, and others.

The first dentist in the United States, of whom we have any account, was Mr. R Wooffendale, who came over from England in the year 1766. Mr. John Greenwood, however, was the first native American dentist. He practiced in New York about the year 1778.

Not until about 1820 do we find much progress in the science in the United States. From that period the advancement of dentistry has been marked. The first college of Dental Surgery was established in Baltimore in 1840. Since then, colleges have been established in all parts of the United States, leading the world in dental educational facilities.

This important branch of medicine has made especially rapid strides during the last two decades. Many inventions and improved instruments, have rendered easy the oftimes tedious and painful operations, these advantages with the intelligence and skill which may be now found in the profession, fully meet the demand for dental operations to prevent the loss of the important organs of mastication. The principle cause of the loss of teeth is pure neglect on the part of their owners. We Americans have worse teeth than the people of most other nations, simply because we do not take the time and trouble to consult a competent dental surgeon. We forget that we have teeth until an exposed pulp in some aching molar cries out for protection, then, when too late perhaps, we look for our friend the Dentist, and the chances are that the forceps have to be used, when if we had taken a little precaution the tooth might have been saved. Many persons seem to think that artificial teeth are "just as good" as those given by nature. I have only to say, try them. Hence we should not neglect the natural teeth thinking that we can easily have them replaced.

